# NRN1 as a therapeutic target for Alzheimer's disease

**DOI:** 10.1002/alz.71149

**Published:** 2026-02-06

**Authors:** Derian A. Pugh, Gregory A. Cary, Kelsey M. Greathouse, Lauren C. Nassour‐Caswell, Emma L. Hobby, Nicholas T. Seyfried, Jeremy H. Herskowitz

**Affiliations:** ^1^ Department of Neurology, Killion Center for Neurodegeneration and Experimental Therapeutics University of Alabama at Birmingham School of Medicine Birmingham USA; ^2^ The Jackson Laboratory Bar Harbor ME USA; ^3^ Department of Biochemistry, Emory Goizueta Alzheimer's Disease Research Center Emory University School of Medicine Atlanta USA

**Keywords:** aging, Alzheimer's disease, antibody, cognition, neurotrophic factor, resilience, Therapeutic Assessment

## Abstract

**INTRODUCTION:**

Neuritin‐1 (NRN1) was identified as a synaptic protein associated with cognitive resilience to Alzheimer's disease (AD).

**METHODS:**

Target risk score and cell type expression profiles were generated for NRN1 using methods developed by the Emory‐Sage‐SGC‐JAX Target Enablement to Accelerate Therapy Development for Alzheimer's Disease (TREAT‐AD) Center and Seattle Alzheimer's Disease Brain Cell Atlas (SEA‐AD). Antibody characterization was conducted using Western blots and densitometry to assess the relative protein abundances of NRN1 in rodents, humans, and cell models.

**RESULTS:**

NRN1 has a TREAT‐AD target risk score of 3.29 out of 5. Based on single‐nucleus RNA sequencing from SEA‐AD, NRN1 expression in excitatory neurons tends to decrease with increasing donor pseudo‐progression. Abcam ab64186 polyclonal NRN1 antibody detects NRN1 protein in vitro and in vivo at molecular weights that suggest NRN1 forms a homodimer. NRN1 protein abundance is comparable among controls and primary tauopathy cases, as well as Tau P301S mice and non‐transgenic littermates at 3 and 9 months.

**DISCUSSION:**

These findings advance the investigation of NRN1 as a therapeutic candidate for AD.

## Highlights

- NRN1 is associated with cognitive resilience to AD.
- Based on snRNA‐seq from SEA‐AD, NRN1 expression in excitatory neurons tends to decrease with increasing donor pseudo‐progression.
- Abcam ab64186 polyclonal NRN1 antibody detects NRN1 protein in vitro and in vivo at molecular weights that suggest NRN1 forms a homodimer.
- NRN1 protein abundance is comparable among controls and primary tauopathy cases, as well as Tau P301S mice and non‐transgenic littermates at 3 and 9 months.

## 1 BACKGROUND

Approximately one‐third of older individuals harbor high levels of amyloid beta (Aβ) and neurofibrillary tangle (NFT) pathology in their brains at autopsy but show little to no signs of cognitive impairment in their lifetime.^1^, ^2^ These individuals who evade cognitive decline despite high levels of Aβ and NFT pathology appear to exhibit cognitive resilience to the clinical manifestations of AD.^3^, ^4^, ^5^ Studies have identified several factors that contribute to the resilience to AD neuropathology, including the ability to maintain brain synapse structure and functional connectivity, potentially through the maintenance of neurotrophic factors.^6^, ^7^, ^8^, ^9^, ^10^, ^11^ Gaining a better understanding of the mechanisms that confer protection against cognitive decline in these resilient individuals could provide the key to preventing or delaying the onset of dementia in susceptible individuals.

To this end, proteomic studies utilizing *post mortem* human brain samples have enabled the quantification of thousands of proteins, fostering a revolution in omics‐based identification of candidate biomarkers and putative therapeutic targets for AD.^12^, ^13^, ^14^, ^15^, ^16^, ^17^, ^18^ While these analyses provide vast lists of molecules that are altered in human disease, few studies demonstrate functional validation of individual proteins nominated as drug targets or biomarkers. Notably, Yu et al. sought to identify proteins associated with cognitive resilience via a proteome‐wide association study of human dorsolateral prefrontal cortex tissue samples obtained from participants of the Religious Orders Study and the Rush Memory and Aging Project (ROSMAP). Eight cortical proteins were identified in association with cognitive resilience, of which Neuritin‐1 (NRN1) was the strongest signal associated with greater resilience.^18^ Specifically, when comparing older persons from ROSMAP with the same age, sex, educational level, and neuropathologic burden, those with higher levels of NRN1 had slower rates of cognitive decline than older persons with lower levels.^18^ Hurst and Pugh et al. sought to extend these findings by conducting multiplex tandem mass tag mass spectrometry (TMT‐MS)‐based proteomics on matched brain tissue from premotor cortex and occipital temporal gyrus, followed by consensus‐weighted gene correlation network analysis. NRN1 was identified as a hub protein in a synaptic co‐expression module that remained increased in resilient individuals compared to symptomatic cases.^9^ Furthermore, NRN1 protein abundance correlated positively with global cognitive performance and cognitive trajectory.^9^ To validate the systems‐level analysis, primary cultured neurons were used to evaluate the neuroprotective mechanisms of NRN1 against Aβ.^9^ Collectively, these studies provided a valuable framework for investigating the molecular and physiological underpinnings of resilience directly from patient samples and cognitive changes in life.

NRN1, also known as candidate plasticity gene 15 (*CPG15*), is a neurotrophic factor that was initially discovered in a screen to identify genes involved in activity‐dependent synaptic plasticity in the rat dentate gyrus.^19^ Since its discovery nearly two decades ago, the role of NRN1 in regulating neurodevelopment and the formation of axonal arbors and dendritic branching has been studied extensively.^20^, ^21^, ^22^, ^23^, ^24^, ^25^, ^26^ In the adult brain, NRN1 is correlated with synapse maturation and long‐term stability, as well as activity‐related plasticity.^19^, ^23^, ^24^, ^25^, ^26^, ^27^, ^28^ However, further studies investigating NRN1's mode of action in AD have been limited in part due to the lack of reliable tools to measure the abundance and function of endogenous proteins.

In this study, we advance the investigation of NRN1 as a therapeutic candidate for AD by evaluating target risk assessment and identifying cell‐type‐specific expression profiles through collaborative studies with the Emory‐Sage‐SGC‐JAX Target Enablement to Accelerate Therapy Development for Alzheimer's Disease (TREAT‐AD) Center. Furthermore, we characterize commercially available polyclonal NRN1 antibodies using in vitro and in vivo model systems as well as *post mortem* human brain tissue samples, providing researchers with a useful tool to further investigate NRN1 protein and function.

## 2 METHODS

### 2.1 Human subjects

*Post mortem* dorsolateral prefrontal cortex tissues from control (*n* = 12), progressive supranuclear palsy (PSP) (*n* = 7), and corticobasal degeneration (CBD) (*n* = 9) cases were selected for comparison from the Emory University Alzheimer's Disease Research Center brain bank. The PSP and CBD cases in this study underwent extensive neuropathological characterization required for diagnosis based on established criteria.^29^, ^30^ Cases were matched as closely as possible for age at death, gender, and *post mortem* interval (Table S1). A single small piece (∼100 mg) was obtained from each brain sample using a sterile razor blade and thawed in 5X lysis buffer (0.5% Nonidet P‐40, 0.5% deoxycholate, 150 mm sodium chloride, and 50 mm Tris, pH 7.4) diluted to 1X in PBS (0.1% Nonidet P‐40, 0.1% deoxycholate, 30 mm sodium chloride, and 10 mm Tris, pH 7.4) with protease and phosphatase inhibitors. Samples were Dounce homogenized and incubated on ice for 20 min. Samples were then centrifuged at 497 × g for 10 min at 4°C. The resulting supernatant was saved and stored at −80°C.

### 2.2 Animals

All experimental procedures were performed under a protocol approved by the Institutional Animal Care and Use Committee (IACUC) at the University of Alabama at Birmingham (UAB). C57BL/6N mice were aged to 3 months and kept on a 12‐h light/dark cycle. Mice were housed five to seven per cage and had ad libitum access to food and water. Both male and female mice were used, and no statistically significant differences in sex were observed.

Tau P301S (Line PS19) mice were purchased from the Jackson Laboratory (B6;C3‐Tg(Prnp‐MAPT*P301S)PS19Vle/J, stock no. 008169) and bred with non‐transgenic (NTG) mice on a B6C3 background. The PS19 mouse line overexpresses 1N4R human tau with the frontotemporal dementia‐associated P301S mutation under the control of the mouse prion protein promoter.^31^ PS19 mice and NTG littermate controls were aged to 3 or 9 months. A total of 24 mice were used, with a sample size of 12 mice per age group and genotype. Both male and female mice were used.

**RESEARCH IN CONTEXT**

**Systematic review**: NRN1 was identified as a top protein candidate associated with cognitive resilience to AD. Previous studies reported that older individuals with higher levels of NRN1 exhibited slower rates of cognitive decline than older individuals with lower levels. Currently, the role of NRN1 in AD pathogenesis remains unknown.

**Interpretation**: Our findings reveal that decreased expression of NRN1 mRNA and protein abundance in AD is likely due to a reduction of NRN1 expression in excitatory neurons. Furthermore, these results provide evidence in mice, humans, and cell models that NRN1 protein exists predominantly as a homodimer.

**Future directions**: Collectively, these findings underscore the need for new research tools and technologies to validate and advance NRN1 into the next phase of therapeutic target development for AD. Further mechanistic studies are necessary at the basic science level to expand these findings and interrogate the putative impact of NRN1 on AD progression.

### 2.3 Perfusions and brain tissue processing

Mice were anesthetized with Fatal Plus and transcardially perfused with cold 1X PBS for 2 min. Immediately following perfusion, each brain was extracted and dissected into two hemispheres. Both hemispheres were flash‐frozen in 2‐methylbutane (Catalog No.: 320404, Sigma‐Aldrich) and sub‐dissected to obtain the prelimbic medial prefrontal cortex and hippocampus. Samples were thawed in 5X lysis buffer (0.5% Nonidet P‐40, 0.5% deoxycholate, 150 mm sodium chloride, and 50 mm Tris, pH 7.4) diluted to 1X in PBS (0.1% Nonidet P‐40, 0.1% deoxycholate, 30 mm sodium chloride, and 10 mm Tris, pH 7.4) with protease and phosphatase inhibitors. Samples were Dounce homogenized and incubated on ice for 20 min. Samples were then centrifuged at 497 × g for 10 min at 4°C. The resulting supernatant was saved and stored at −80°C.

### 2.4 Cell lines

Human embryonic kidney (HEK) 293T cell lines (Catalog No.: CRL‐3216, ATCC) were used. Cells were maintained in DMEM: high glucose, pyruvate (Catalog No.: 11995073, Invitrogen) + 10% fetal bovine serum (FBS) (Catalog No.: 35016CV, Corning) + 1% penicillin‐streptomycin (Catalog No.: 15140122, Invitrogen) at 37°C with 5% CO_2_. Neuro‐2a (N2a) mouse neuroblastoma cell lines (Catalog No.: CCL‐131, ATCC) were maintained in MEM (Catalog No.: 11095‐080, Thermo Fisher Scientific) with 10% FBS and 1% penicillin‐streptomycin. SH‐SY5Y human neuroblastoma cell lines (Catalog No.: CRL‐2266, ATCC) were maintained in a 1:1 mixture of DMEM/F12 (Catalog No.: 11330057, Thermo Fisher Scientific) and Eagle's Minimum Essential Medium (Catalog No.: 50‐238‐2632, Fisher Scientific) with 10% FBS and 1% penicillin‐streptomycin. All cell lines were used between passages 8 and 20 for all experiments. Three independent cultures of SH‐SY5Y, HEK293T, or N2a cell lines were used per experiment, representing three biological replicates.

### 2.5 Primary neuron cultures

Primary rat neuronal cultures were generated from E18 Sprague‐Dawley rat embryos as previously described.^9^, ^32^, ^33^ Rats were euthanized with procedures that are consistent with the recommendations of the American Veterinary Medical Association Guidelines for the Euthanasia of Animals and approved by the UAB IACUC. Briefly, cell culture plates were coated overnight with 1 mg/mL poly‐L‐lysine (Catalog No.: P2636‐100MG, Sigma–Aldrich) and rinsed with diH_2_O. Neurons were cultured at a density of 4 × 10^5^ cells per well on a 12‐well plate. Neurons were cultured in Neurobasal medium (Catalog No.: 21103‐049, Thermo Fisher Scientific) supplemented with B27 (Catalog No.: 17504‐044, Thermo Fisher Scientific). Cultures received half‐medium changes every 3 days in vitro (DIV) until DIV 14, when cells were processed for Western blotting.

### 2.6 Chemicals and reagents

2‐Mercaptoethanol (BME) (Thermo 1610710, Bio‐Rad) was prepared according to the instructions of the manufacturer. Dithiotheitol (DTT) (Thermo R0861, Thermo Fisher Scientific) sample buffer was made by combining 1M DTT with 2X Laemmli buffer (Thermo Fisher Scientific 1610737, Bio‐Rad), for a final concentration of 50 mM DTT. Samples were boiled at 95°C for 5 min. DTT + Urea (Catalog No.: U5128‐500G, Sigma‐Aldrich) sample buffer was made by combining 1M DTT and 8M Urea in 2X Laemmli buffer, for a final concentration of 50 mM DTT + 6M Urea. Samples were incubated at room temperature for 10 min. Bond‐Breaker TCEP Solution (Catalog No.: 77720, Thermo Fisher Scientific) in 2X SDS sample buffer was made by combining 0.5M TCEP with 2X SDS for a final concentration of 50 mM TCEP. Samples were boiled at 95°C for 5 min. Bond‐Breaker TCEP Solution in Laemmli sample buffer made by combining 0.5M TCEP with 2X Laemmli buffer for a final concentration of 50 mM TCEP. 2X SDS sample buffer was made by combining 10% SDS (Catalog No.: BP166‐500, Thermo Fisher Scientific), 0.5M Tris HCl pH 6.8 (Catalog No.: H5121, Promega), glycerol (Catalog No.: 158922500, Thermo Fisher Scientific), 0.05% Bromophenol Blue (Catalog No.: BP114‐25, Thermo Fisher Scientific), 2‐Mercaptoethanol (Catalog No.: 1610710, Bio‐Rad), and diH_2_O. Samples were boiled at 95°C for 5 min. All boiled samples were allowed to cool before loading into gels.

### 2.7 NRN1 siRNA knockdown

Neuro‐2a (N2a) mouse neuroblastoma cell lines (Catalog No.: CCL‐131, ATCC) were seeded at a density of 4 × 10^5^ onto 12‐well plates and were allowed to adhere overnight. The next day, the following small interfering RNAs (siRNAs) were reconstituted in UltraPure DNase/RNase‐Free Distilled Water (Catalog No.: 10977‐015, Invitrogen) to make 20 µM stocks: ON‐TARGETplus NRN1 siRNA (Catalog No.: L‐040328‐01‐0005, Horizon) and ON‐TARGETplus Non‐targeting Pool (Catalog No.: D‐001810‐10‐05, Horizon). The comprehensive transfection reagent was made per the manufacturer's instructions (Horizon), as follows. Label two separate tubes for each condition. In tube 1, combine 5 µM siRNA with MEM medium (Catalog No.: MT10010CV, Corning) for a total volume of 100 µL per well. In tube 2, combine 4 µL DharmaFECT 1 Transfection Reagent (Catalog No.: T‐2001‐01, Horizon) with MEM medium for a total volume of 100 µL per well. Allow tubes to incubate for 5 min at room temperature. Combine tubes 1 and 2 and incubate for 20 min at room temperature. Add 800 µL of MEM medium to the tube containing tubes 1 and 2 for a final volume of 1 mL per well. Transfection reagent cocktails were added dropwise to each well containing 500 µL of existing medium and allowed to incubate at 37°C for 6 h before the medium was changed. Cells were then incubated at 37°C for 96 h before collection, with daily medium changes.

### 2.8 Harvesting cells for Western blot

Twelve‐well culture plates were removed from the incubator and placed on ice. Media were aspirated from each well. One milliliter 1X PBS with protease and phosphatase inhibitors was added to each well. Wells were scraped with a cell scraper, and contents were transferred to a 1.5‐mL tube. Tubes were centrifuged at 497 × g for 5 min at 4°C. The supernatant was discarded and pellets were resuspended in lysis buffer diluted to 1X in PBS with protease and phosphatase inhibitors. 5X lysis buffer: 0.5% Nonidet P‐40, 0.5% deoxycholate, 150 mm sodium chloride, and 50 mm Tris, pH 7.4. The 1X lysis buffer consisted of 0.1% Nonidet P‐40, 0.1% deoxycholate, 30 mm sodium chloride, and 10 mm Tris, pH 7.4. Samples were vortexed and incubated on ice for 20 min. Samples were then centrifuged at 497 × g for 10 min at 4°C. The resulting supernatant was saved and stored at −80°C.

### 2.9 Western blot and antibodies

Protein concentration was determined by bicinchoninic acid (BCA) protein assay (Catalog No.: FERA65453, Thermo Fisher Scientific). Samples were added to loading dye to yield the desired amount of protein in a final volume of 30 µL. The samples were then boiled at 95°C for 4 min, spun down briefly in a benchtop centrifuge, then loaded on precast 4% to 15% gradient gels (Catalog No.: 5671084, Bio‐Rad). Blots were run for 20 min at 50 V, then 40 to 45 min at 200 V. A 10X transfer buffer was prepared containing 0.2M Tris based and 1.53M glycine (pH 8.1to8.5). The buffer was diluted to 1X (20 mM Tris base, 153 mM glycine) with deionized water before use. Samples were then transferred from the polyacrylamide gel to a polyvinylidene fluoride membrane (Catalog No.: 1620264, Bio‐Rad) at a constant 0.10 A and 6 V for 16 h. Membranes were blocked in 1X Casein Blocking Buffer (Catalog No.: B6429, Sigma‐Aldrich) at room temperature for 1 h, then incubated in primary antibody overnight at 4°C. The next day, membranes were washed in 1X TBS + 0.1% Tween for 5 min at room temperature, followed by two 5‐min washes in 1X TBS. Membranes were then incubated in secondary antibody for 1 h at room temperature, followed by another set of washes. Images were captured using an Odyssey Image Station (LI‐COR Biotechnology), and band intensities were quantified using ImageStudio Lite software version 5.2 (LI‐COR Biotechnology).

Primary antibodies: anti‐NRN1 rabbit polyclonal (Catalog No.: ab64186, Abcam, RRID: AB_2236282), anti‐NRN1 rabbit polyclonal (Catalog No.: bs‐2464R, Bioss, RRID: AB_10886278), anti‐NRN1 rabbit polyclonal (Catalog No.: PA5‐47406, Invitrogen, RRID: AB_2577114), glyceraldehyde‐3‐phosphate dehydrogenase (GAPDH) mouse monoclonal (Catalog No.: MAB374, Millipore Sigma, RRID: AB_2107445), Myc‐tag (71D10) rabbit monoclonal (Catalog No.: 2278S, Cell Signaling, RRID: AB_490778). Secondary antibodies: IRDye 800CW goat anti‐mouse IgG secondary antibody (Catalog No.: 926‐32210, LI‐COR Biosciences, RRID: AB_621842), goat anti‐rabbit IgG (H+L) highly crossed‐adsorbed secondary, Alexa Fluor 680 (Catalog No.: A‐21109, Thermo Fisher Scientific, RRID: AB_2535758).

### 2.10 Human iPSC‐derived cell cultures

ioAstrocytes (Catalog No.: ioEA1093, bit.bio) and ioGlutamatergic neurons (Catalog No.: Io1001S, bit.bio) were cultured exactly as described in bit.bio protocols (www.bit.bio/resources/protocols/human‐ipsc‐derived‐astrocytes‐glutamatergic‐co‐culture‐protocol). The 18‐mm glass coverslips (Catalog No.: 64‐0714, Warner Instruments) were cleaned as previously described.^33^ Coverslips were coated with poly‐d‐lysine (Catalog No.: P6407, Sigma‐Aldrich) for 3 h and Geltrex (Catalog No.: A1413202, Thermo Fisher Scientific) for 1 h, using concentrations in bit.bio protocols. ioAstrocytes were plated at 114,000 cells per well for immunocytochemistry or biochemistry, on coverslips or in 12‐well tissue culture plates, respectively. Nine days later, ioGlutamatergic neurons were plated on top of the established astrocytes at a density of 114,000 cells per coverslip or well. Medium formulations and medium changes were performed precisely as described, with no modifications in the bit.bio protocol, according to the link above.

### 2.11 Immunocytochemistry and antibodies

Coverslips were fixed and stained as previously described,^34^, ^35^, ^36^ with minor modifications. Coverslips were fixed at DIV 23 for 15 min in fresh 4% paraformaldehyde (Catalog No.: P6148, Sigma‐Aldrich). Coverslips were rinsed three times in PBS containing 0.5% normal horse serum, 0.5% normal goat serum, and 0.05% saponin (wash buffer). Next, coverslips were blocked and permeabilized for 30 min with PBS containing 5% normal horse serum, 5% normal goat serum, 1% bovine serum albumin, and 0.05% saponin (blocking buffer) plus 0.05% Triton X‐100 at room temperature. Primary antibodies were diluted 1:100 and incubated overnight at 4°C. Coverslips were subsequently rinsed three times in wash buffer and incubated in secondary antibodies at 1:100 for 1 h at room temperature in blocking buffer. Coverslips were rinsed three times in wash buffer, and the last rinse included 5 µL of DAPI (4′,6‐diamidino‐2‐phenylindole) (Catalog No.: D1306, Thermo Fisher Scientific). Coverslips were mounted on slides in Vectashield (Catalog No.: H‐1000, Vector Laboratories), sealed with nail polish, and stored at 4°C. Primary antibodies: anti‐MAP2 chicken polyclonal (PA1‐10005, Millipore Sigma, RRID: AB_1076848), anti‐S100β rabbit monoclonal (Catalog No.: ab52642, Abcam), anti‐NRN1 rabbit polyclonal (ab64186, Abcam, RRID: AB_2236282). Secondary Antibodies: AlexaFluor 488 goat anti‐chicken (Catalog No.: A11039, Invitrogen, RRID: AB_2534096), AlexaFluor 594 goat anti‐rabbit (Catalog No.: A11037, Invitrogen, RRID: AB_2534095).

### 2.12 Confocal microscopy

Confocal microscopy was used to capture images of ioAstrocytes and ioGlutamatergic neurons. Our methods are based on previously described methods.^36^ Briefly, microscopy was performed on a Nikon (Tokyo, Japan) Ti2 C2 confocal microscope, using Plan Apo 20×/0.75 NA air objective and Plan Apo 60×/1.40 NA oil‐immersion objective. Three‐dimensional z‐stacks were obtained. Nikon Elements 4.20.02 image capture software was used to acquire z‐stacks at 60× magnification with a step size of 0.1 µm, image size of 1024 × 1024 pixels, and acquisition rate of one frame/s. Nikon Elements 4.20.02 image capture software was used to acquire z‐stacks at 20× magnification with a step size of 0.85 µm, image size of 1024 × 1024 pixels, and acquisition rate of one frame/s.

### 2.13 Statistical analysis

Statistical analyses were conducted using GraphPad Prism 10.4.2 (GraphPad Software, La Jolla, CA, USA). Specifics of each statistical test are provided in the figure legends. Data are presented as mean ± standard deviation (SD), and all graph error bars represent SD. Significance was defined as *p *< 0.05. See figure legends for details on *N* per experiment. Figures were assembled and generated in Adobe Illustrator (version 29.4).

### 2.14 TREAT‐AD target risk score

AD relevance was assessed using an integrated measure of disease association developed by the Emory‐Sage‐SGC‐JAX TREAT‐AD Center.^37^ The target risk score compiles evidence for each gene in the genome association with AD phenotypes using genetic association studies as well as *post mortem* transcriptomic and proteomic assessment of brains from AD and non‐demented control individuals. The target risk score is the sum of the target's Genetics Score and Genomics Score. Target risk score values range from 0 to 5, with 5 being evidence of the strongest association with AD. These scores were downloaded from the Synapse platform (syn25741025) and are accessible in supplements from the publication describing the generation of these metrics.^37^

### 2.15 SEA‐AD cell‐type expression profile

Cell‐type expression profiles were determined using single‐nucleus RNA sequencing (snRNA‐seq) data from the Seattle Alzheimer's Disease Brain Cell Atlas (SEA‐AD) (sea‐ad.org). SEA‐AD has generated and made fully available snRNA‐seq data from the medial temporal gyrus of individual donors over a range of neuropathological and clinical statuses. Individual donors were stratified using a pseudo‐progression score learned from composite representations of extensive neuropathological and cellular characterization of donor tissues, described in detail in the associated publication.^38^ The processed data (“SEAAD_MTG_RNAseq_final‐nuclei.2024‐02‐13.h5ad”) were downloaded from the links available at the study page, and the expression from each donor was averaged across all cells within a particular supertype classification. Locally Weighted Scatterplot Smoothing (LOWESS) was applied to donor‐level mean expression values of supertypes within each subclass to visualize the trend across the range of continuous donor pseudo‐progression.^39^

### 2.16 Gene set enrichment analysis (GSEA) and biodomain assessment

Proteomic results were accessed from the publication.^9^ Significantly enriched Gene Ontology (GO) terms were identified with GSEA in R (version 4.4.3) that was performed using the gseGO function from the clusterProfiler R package (version 4.14.6),^40^ which utilized the org.Rn.eg.db annotation database (genome‐wide annotation for rat, R package version 3.19.1). The log2 fold‐change values of all proteins comparing NRN1 treatment versus vehicle control were used as the GSEA ranking statistic. Significantly enriched GO terms (false discovery rate [FDR] ≤ 0.05) were then mapped to the biological domains (biodomains) of AD,^37^ which are available via the Synapse platform (syn25428992). Results were compared with GSEA meta‐analysis of human brain proteomic data from the Accelerating Medicines Partnership Program for Alzheimer's Disease (AMP‐AD) studies.^37^

## 3 RESULTS

### 3.1 TREAT‐AD target ranking and SEA‐AD cell‐type expression profiles for NRN1

**FIGURE 1:**
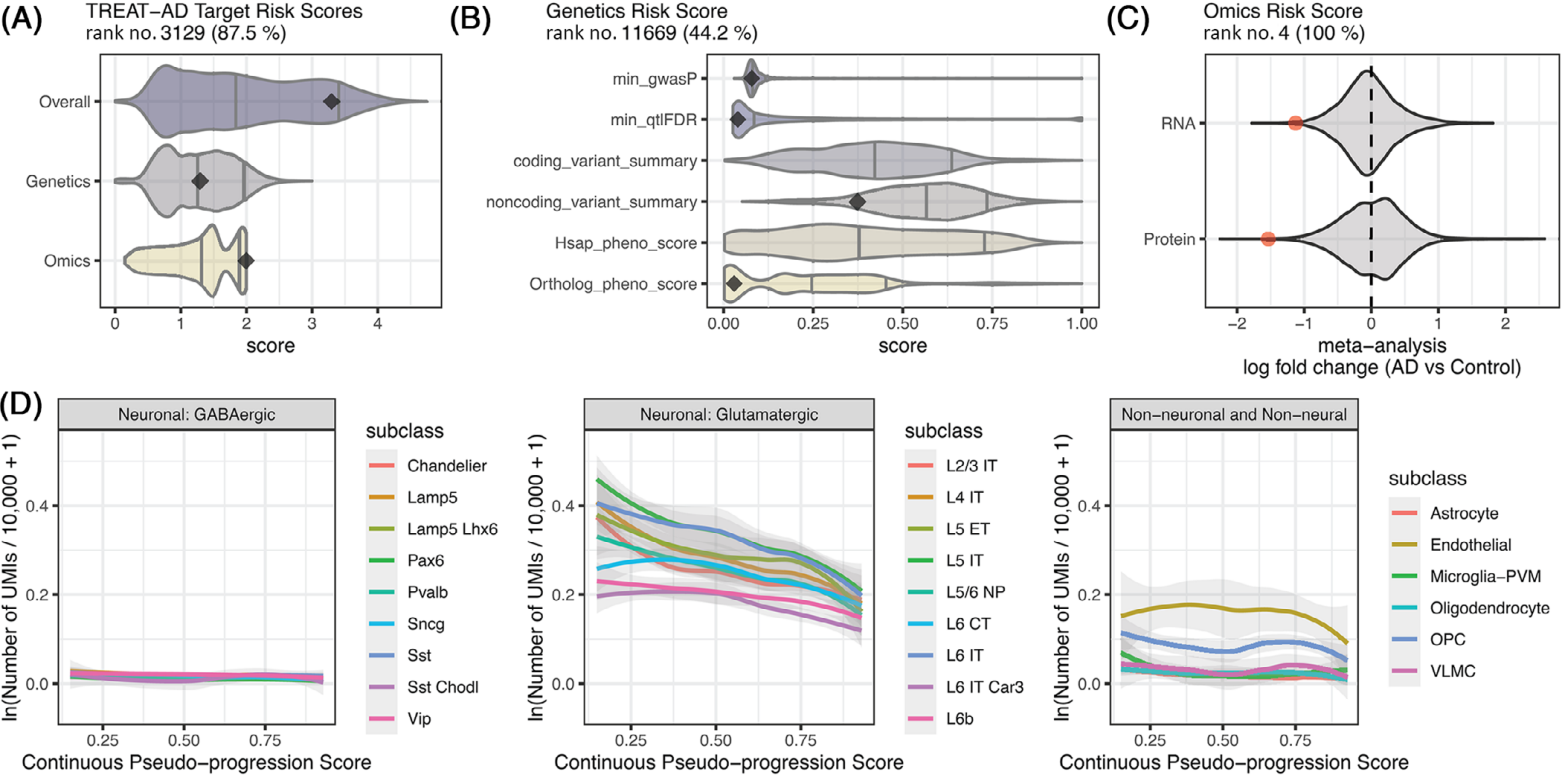
TREAT‐AD target risk scores and SEA‐AD cell type expression profiles for NRN1. (A) Violin plots show the distribution of all Target Risk Scores (i.e., overall), as well as the component scores for genetics and genomics. The score for NRN1 in each case is shown as a point. (B) Evidence feeding genetic risk score, including minimum GWAS *p* value across studies (min_gwasP), minimum xQTL FDR across studies (min_qtlFDR), summary assessments for identified coding and non‐coding variants assigned to NRN1, metrics for overlapping phenotypes from human gene annotations (Hsap_pheno_score), and phenotypes annotated to orthologous genes (Ortholog_pheno_score). (C) Summary of meta‐analysis results for AMP‐AD transcriptomics and proteomics data showing the distribution of fold‐change values for all genes and proteins, as well as where NRN1 is located within those distributions. (D) Locally weighted mean expression of all cell supertypes within a given subtype from donors across the continuous donor pseudo‐progression. Higher pseudo‐progression scores correspond to more AD clinicopathologic traits in donors. AD, Alzheimer's disease; AMP‐AD, Accelerating Medicines Partnership Program for Alzheimer's Disease; FDR, false discovery rate; GWAS, genome‐wide association study; NRN1, Neuritin‐1; SEA‐AD, Seattle Alzheimer's Disease Brain Cell Atlas; TREAT‐AD, Target Enablement to Accelerate Therapy Development for Alzheimer's Disease; xQTL, Quantitive Trait Locus.

The Emory‐Sage‐SGC‐JAX TREAT‐AD Center has developed an integrative target ranking score encompassing evidence of gene and protein involvement in AD from both genomics and genetics studies.^37^ NRN1 has a TREAT‐AD target risk score of 3.29 out of 5 (rank 3129, 87.5th percentile) (Figure 1A). The risk score components indicate a relatively stronger disease association based on genomics from *post mortem* human brain tissue samples compared to genetic studies, including 1.29 out of 3 for genetic risk and 1.99 out of 2 for genomic risk. Across genome‐wide association studies, there are no single‐nucleotide polymorphisms near NRN1 that reach genome‐wide significance and only limited overlap between phenotypes annotated to either AD [MONDO:0004975] or dementia [MONDO:0001627] and phenotypes attributed to NRN1 or NRN1 orthologs (Figure 1B). The meta‐analysis of proteomic and transcriptomic data used to compute the genomics risk score indicates that the NRN1 protein abundance and RNA expression are decreased significantly in brains from patients with AD (Figure 1C). Cell‐type‐specific RNA expression of NRN1 was assessed using single‐cell data from SEA‐AD (sea‐ad.org). The expression of NRN1 was averaged across cells within a supertype from each donor. LOWESS was applied to donor‐level mean expression values of supertypes within each subclass to visualize the trend across the range of continuous donor pseudo‐progression, which orders subjects based on a learned model of neuropathological burdenm.^38^ NRN1 expression is highest in excitatory neurons with very limited expression in inhibitory neuron subclasses (Figure 1D). NRN1 expression in excitatory neurons tends to decrease with increasing donor pseudo‐progression. NRN1 expression is also detected in endothelial cells and oligodendrocyte precursor cells in the SEA‐AD dataset, but NRN1 expression is relatively stable in these cell types across donor pseudo‐progression. While decreased levels of NRN1 in bulk proteomics and transcriptomics could be in part due to neuronal loss, the single‐cell transcriptomic analyses suggest that excitatory neurons that remain at later stages of disease progression express lower levels of NRN1 than excitatory neurons from brains at earlier stages of AD.

### 3.2 Comparative biodomain assessment of cultured neurons treated with NRN1 and AD brains

**FIGURE 2:**
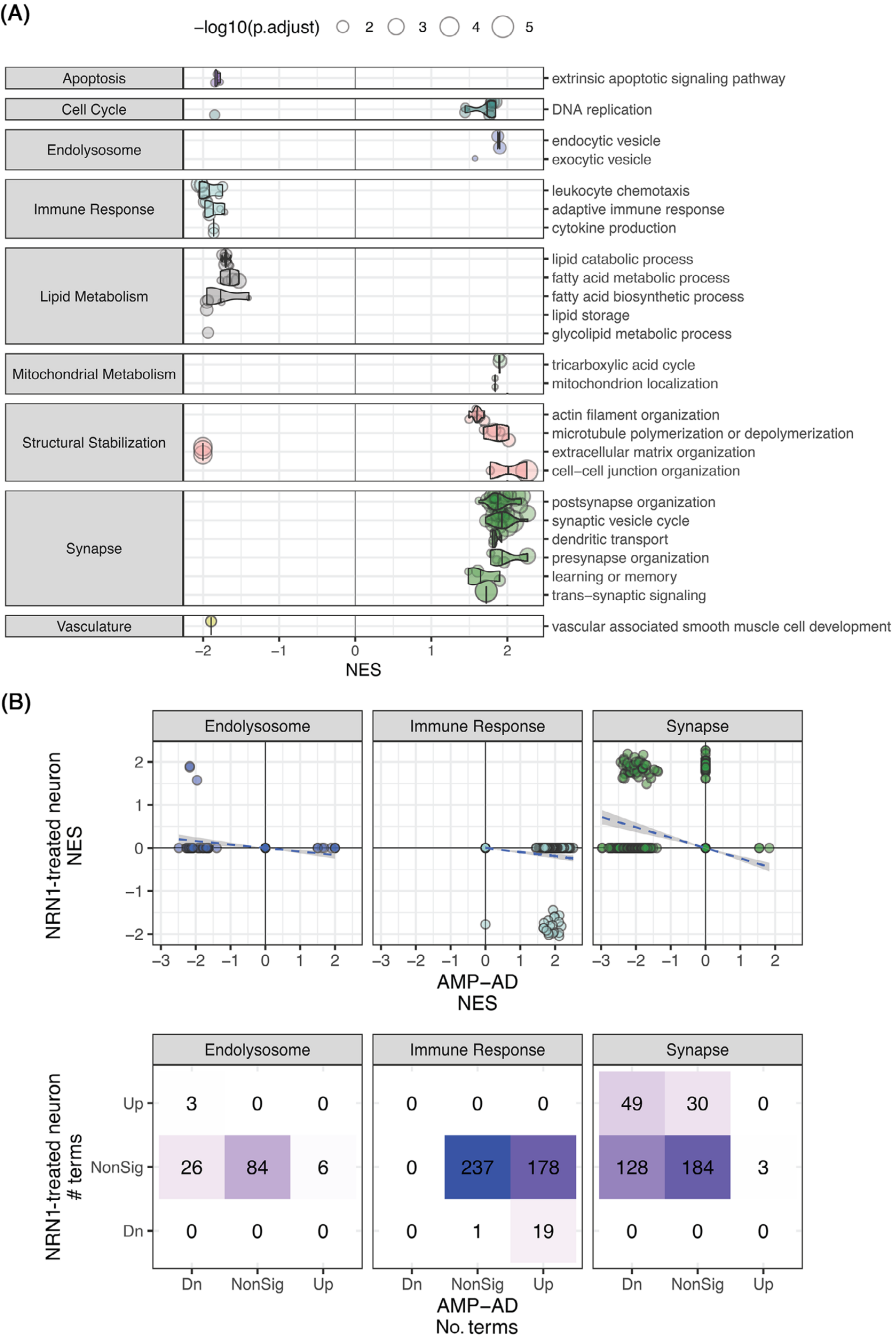
Proteomic effects of NRN1 treatment on primary cultured rat cortical neurons. (A) Gene set enrichment results of proteomics from primary cultured rat cortical neurons exposed to NRN1 or vehicle control. Each point is a GO term mapped within a subgroup of terms from each identified biodomain. The size of each point corresponds to the significance of the enrichment. The position along the *x*‐axis (i.e., NES) corresponds to whether proteins within that term are found to be up‐ or downregulated following NRN1 treatment. (B) Comparison between GSEA results from NRN1‐treated rat primary neurons and postmortem AD brains, plotting the number of significantly enriched terms shared by each analysis and whether the terms are enriched in the same or opposing directions. Terms from the Endolysosome, Immune Response, and Synapse domains are plotted (top row) and tabulated (bottom row). GO, Gene Ontology; GSEA, Gene Set Enrichment Analysis; NRN1, Neuritin‐1.

The Emory‐Sage‐SGC‐JAX TREAT‐AD Center developed a target categorization system that is specific to AD‐related processes, termed biological domains (biodomains).^37^ These biodomains are defined by constituent GO terms and allow genes to be annotated to specific biodomains via GO term annotations. NRN1 is annotated to two biodomain terms, “synapse” (GO:0045202) and “neuron projection extension” (GO:1990138), both of which are within the Synapse biodomain. Next, we explored the effects of NRN1 treatment on primary cultured rat neurons using this framework. To accomplish this, we re‐analyzed proteomic measures collected from rat primary cortical neuron cultures treated with either ectopic NRN1 or a vehicle control,^9^ then used GSEA to map significantly enriched GO terms (FDR ≤ 0.05) into biodomains (Figure 2A). The primary effects of NRN1 treatment on the proteome of cultured rat cortical neurons included increased expression of proteins in 79 GO terms from the Synapse biodomain, particularly in the context of “postsynapse organization” and “synaptic vesicle cycle.” Additionally, there were similar increases in proteins from terms within the Mitochondrial Metabolism and Endolysosome biodomains. There were also increases in proteins from 16 terms within the Structural Stabilization biodomain, including cell–cell junction and cytoskeletal‐related terms. In contrast, decreases in proteins from five terms related to the Extracellular Matrix Organization subdomain were identified. We also noted protein decreases following NRN1 treatment from other biodomains, including Immune Response, Lipid Metabolism, and Apoptosis. Next, we compared the experimental rat proteomics GSEA analyses to GSEA results from human brain proteomics studies in the AMP‐AD consortium, which was previously analyzed against the human annotation database.^37^ Comparison of AD and control individuals revealed that many of the terms affected by NRN1 treatment in the primary rat neuron cultures were also affected in AD brains. Almost two‐thirds of the Synapse domain terms that are upregulated following NRN1 treatment (49 of 79 terms) were also found to be enriched among downregulated proteins in AD (Figure 2B). These results corroborate the potential for beneficial therapeutic impact of enhancing NRN1 expression and activity within the brains of AD patients. These findings support prior studies showing that NRN1 can promote changes in vitro that reverse key molecular signatures associated with AD in the human brain.

### 3.3 Characterization of commercially available polyclonal anti‐NRN1 antibodies

**FIGURE 3:**
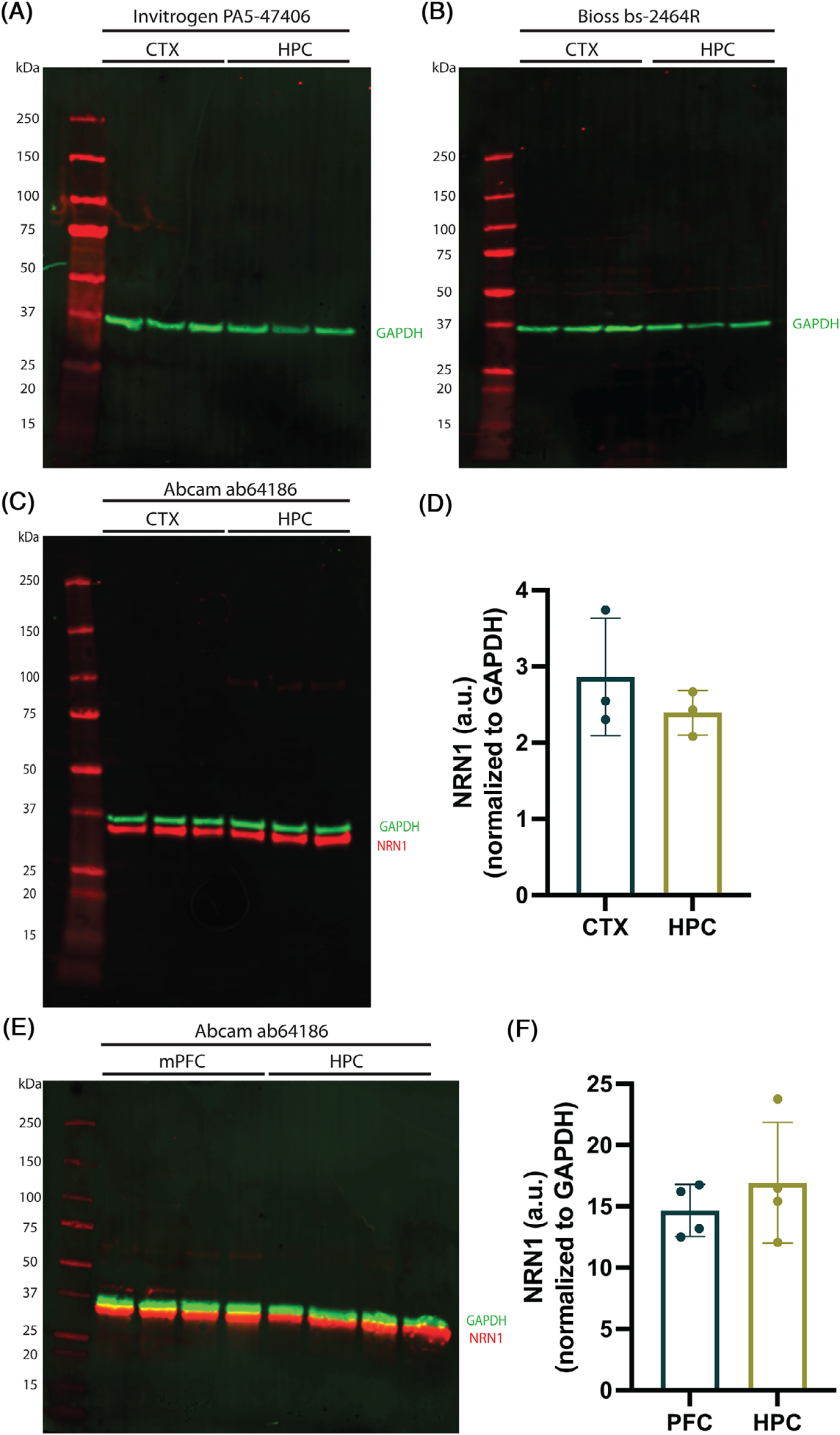
Abcam ab64186 recognizes NRN1 protein in rat primary neurons and mouse brain. (A) Representative Western blot of rat primary cortical (CTX) and hippocampal (HPC) neuron lysates (50 µg protein), probed with NRN1 polyclonal antibody Invitrogen PA5‐47406. (B) Representative Western blot of rat primary CTX and HPC neuron lysates, probed with NRN1 polyclonal antibody Bioss bs‐2464R. (C) Representative Western blot of rat primary CTX and HPC neuron lysates, probed with NRN1 polyclonal antibody Abcam ab64186. (D) Densitometry analysis of panel (C) reveals that when normalized to GAPDH, the observed protein levels of NRN1 are comparable in rat CTX or HPC primary neuron cultures (*t*[4] = 0.9868, *p* = 0.3796). Unpaired *t*‐tests were used for comparisons. (E) Representative Western blot of WT C57BL/6N mice lysate (50 µg protein), probed with NRN1 polyclonal antibody Abcam ab64186. (F) Densitometry analysis of panel (E) reveals that when normalized to GAPDH, the observed protein levels of NRN1 in WT C57BL/6N mice are equivalent in the prelimbic mPFC and hippocampus (HPC) *t*(6 = 0.8453, *p* = 0.4304). Unpaired two‐tailed *t*‐tests were used for comparisons. Each point represents one mouse. *N* = 4 mice (2 male, 2 female). Error bars represent the standard deviation of the mean. a.u. = arbitrary units. GAPDH, glyceraldehyde‐3‐phosphate dehydrogenase; mPFC, medial prefrontal cortex; NRN1, Neuritin‐1; WT, wild type.

Antibodies are among the most frequently used tools in biomedical and clinical research.^41^ The ability to detect changes in protein abundance or molecular weight, localization, or interactions with other proteins is crucial to identify pathways involved in cell regulation and disease pathogenesis. Historically, the availability of specific antibodies for NRN1 has been limited, which in turn has hindered an assessment of endogenous NRN1 protein expression in both normal and disease conditions. However, increasing interest in high‐quality research tools to validate and advance drug targets for AD has recently promoted the expansion of antibodies claimed to react with endogenous NRN1. Unfortunately, many of these antibodies report inconsistencies in the molecular weight of endogenous NRN1.^42^, ^43^, ^44^ To better understand and address the discrepancies in the reporting of endogenous NRN1 protein expression, we conducted a variety of assays on cells and brain tissues to carefully validate antibodies purported to recognize endogenous NRN1. Herein, we compared three commercially available polyclonal (pAb) NRN1 antibodies: Abcam ab64186, Bioss bs‐2464R, and Invitrogen PA5‐47406. NRN1 is highly conserved among vertebrates, exhibiting ∼97% amino acid sequence identity among human, mouse, and rat (Figure S1).^20^, ^45^ According to the product descriptions, Abcam ab64186 recognizes human NRN1; Bioss bs‐2464R recognizes human, mouse, and rat NRN1; and Invitrogen PA5‐47406 recognizes human, mouse, and rat NRN1. Among the commercially available pAb NRN1 antibodies, only Invitrogen PA5‐47406 specifies the immunogen used to generate the antibody‐NRN1 amino acids A28‐T78. Rat primary cortical (CTX) or hippocampal (HPC) neurons were isolated independently at E18 and cultured at high density on a 12‐well culture plate. At 14 DIV, cells were harvested for Western blot and densitometry analysis. Invitrogen PA5‐47406 pAb did not detect endogenous NRN1 protein in cortical or hippocampal cultures (Figure 3A). Bioss bs‐2464R pAb did not detect endogenous NRN1 protein in cortical or hippocampal cultures (Figure 3B). Abcam ab64186 pAb detected a single band at approximately 34 kDa from cortical and hippocampal cultures (Figure 3C). Densitometry analysis revealed that when normalized to glyceraldehyde‐3‐phosphate dehydrogenase (GAPDH), NRN1 protein levels were relatively similar in cortical and hippocampal neuron cultures (Figure 3D).

To test whether the single NRN1 band at ∼34 kDa was exclusive to cultured primary rat neurons, we examined NRN1 protein levels in the prelimbic medial prefrontal cortex (mPFC) and hippocampus (HPC) of 3‐month‐old wild‐type (WT) C57BL/6N mice using Western blot analysis. Abcam ab64186 pAb recognized an ∼34 kDa NRN1 protein band in the prefrontal cortex and hippocampus, which is consistent with the cultured primary neuron experiments (Figure 3E). Densitometry analysis indicated that when normalized to GAPDH, endogenous NRN1 protein levels were not significantly different in the prefrontal cortex when compared to the hippocampus (Figure 3F).

NRN1 is a neurotrophic factor that plays multiple roles in the process of neural development and synaptic plasticity.^45^ Neurotrophins, such as brain‐derived neurotrophic factor (BDNF) and neurotrophin‐3 (NT‐3), can function as non‐covalently associated dimers, which is crucial for biological processes, particularly receptor activation and downstream signaling cascades.^46^ Endogenous NRN1 protein molecular weight is predicted to be 15.3 kDa, based on the putative full‐length amino acid sequence deduced from a cDNA library made from rat hippocampal dentate gyrus.^20^ Based on the ∼34 kDa weight of the observed NRN1 bands in the aforementioned Western blots, we hypothesized that these bands would reflect dimerization of NRN1 monomers. To test this, rat primary cortical neurons were isolated at E18 and cultured at high density on a 12‐well culture plate. At 14 DIV, cultures were harvested for Western blots, which were probed with Abcam ab64186 pAb. To enhance protein denaturing, lysates were treated with fresh preparations of BME and then boiled at 95°C for 4 or 10 min. However, Western blot analyses revealed that combinations of BME and boiling did not collapse the NRN1 band at ∼34 kDa (Figure S2). Next, lysates were treated with fresh preparations of DTT, DTT and Urea, or Tris (2‐carboxyethyl) phosphine (TCEP), then boiled at 95°C for 4 min. Western blot analyses revealed that these treatments did not collapse the NRN1 band at ∼34 kDa (Figure S3). Collectively, these results suggest that if NRN1 exists as a homodimer, the addition of BME, DTT, DTT and Urea, or TCEP is not sufficient to break putative non‐covalent interactions between NRN1 monomers under these experimental conditions. Alternatively, NRN1 may display aggregation artifacts in rat primary neuron cortical cultures or lysates that prevent protein denaturation using these treatments.

### 3.4 Abcam anti‐NRN1 antibody exhibits specificity to NRN1

**FIGURE 4:**
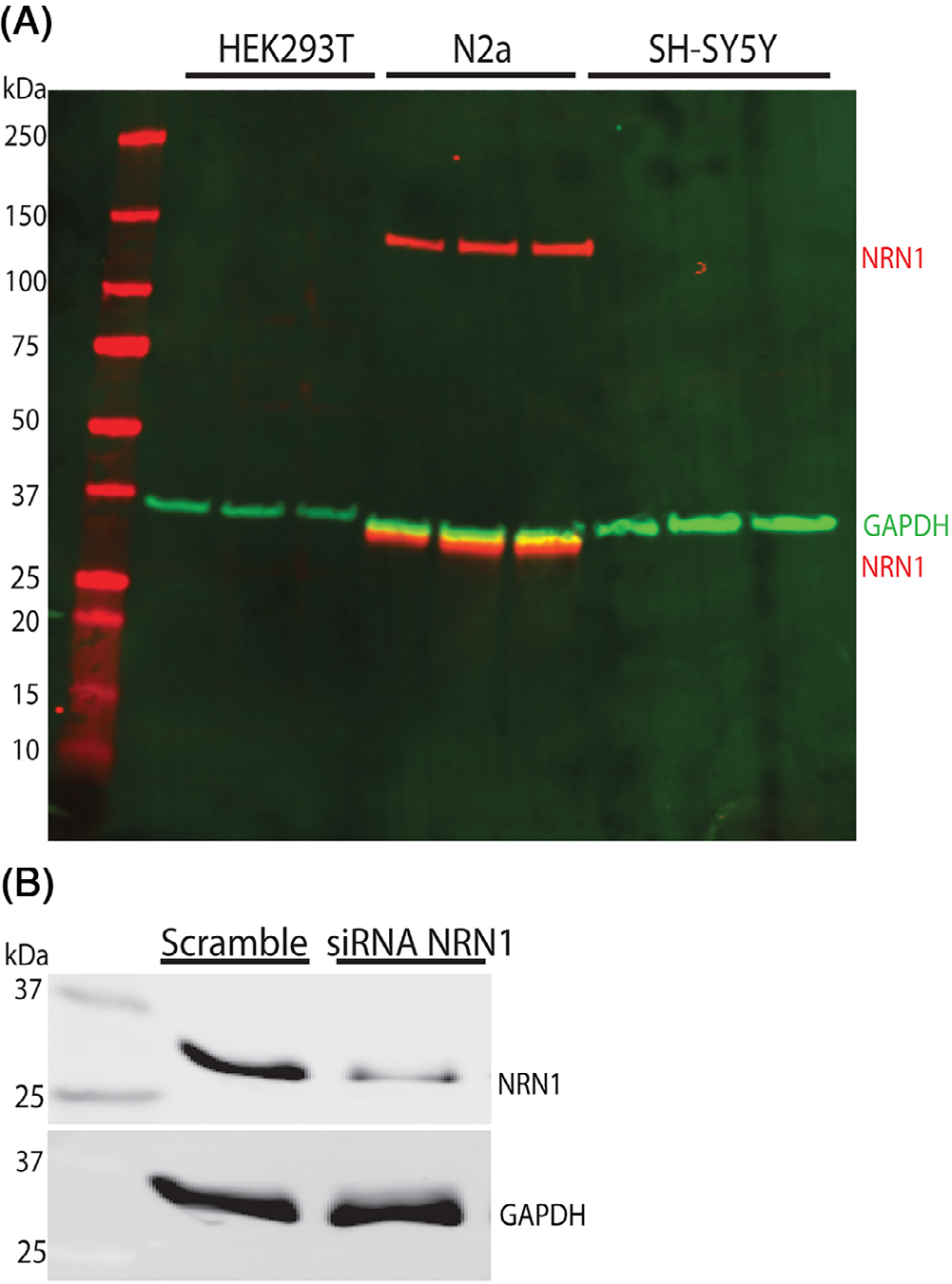
Abcam ab64186 recognizes NRN1 protein in mouse neuroblastoma cells. (A) Representative Western blot of human embryonic kidney (HEK) 293T, Neuro‐2a (N2a) mouse neuroblastoma, and SH‐SY5Y human neuroblastoma cell lysates (50 µg protein), probed with NRN1 polyclonal antibody Abcam ab64186 and GAPDH monoclonal antibody. (B) N2a cells were transfected with NRN1 or Scramble (non‐targeting) siRNA smart pools and harvested after 96 h. Western blot analyses with NRN1 polyclonal antibody Abcam ab64186 revealed reduced intensity of the ∼34 kDa band in NRN1‐depleted cells, compared to Scramble control. 10 µg of protein was loaded per lane, and GAPDH was probed as a loading control. GAPDH, glyceraldehyde‐3‐phosphate dehydrogenase; NRN1, Neuritin‐1; siRNA, small interfering RNA;

Antibody specificity can be assessed by measuring the relevant signal in cells or tissues in which the target molecule is not present.^47^ Previous reports indicated that NRN1 mRNA was found predominantly in the brain, with no detectable expression in the kidney or leukocytes.^20^, ^48^ Based on this, we hypothesized that Abcam ab64186 would not detect endogenous NRN1 protein in HEK 293T or SH‐SY5Y human neuroblastoma cell lines, which putatively lack endogenous NRN1 protein. Moreover, we hypothesized that Abcam ab64186 pAb would detect endogenous NRN protein in Neuro‐2a (N2a) mouse neuroblastoma cell lines, which express endogenous NRN1 protein. To test this hypothesis, HEK293T, N2a, and SH‐SY5Y cells were cultured in 12‐well culture plates. After 72 h, cultures were harvested for Western blotting and densitometry analysis. Whole‐cell lysates were probed using Abcam ab64186. No detectable bands were observed in HEK293T or SH‐SY5Y lysate. However, probing with Abcam ab64186 pAb revealed two bands, at ∼34 kDa and ∼150 kDa, in N2a cells (Figure 4A). These findings suggest that NRN1 homodimers may further associate to form larger macromolecular protein complexes or exhibit aggregation properties, which could be exclusive to N2a cells. To further test the specificity of the Abcam ab64186 pAb, N2a cells were transiently transfected with NRN1‐targeted or scramble siRNA. Ninety‐six hours later, cultures were harvested for Western blotting. Probing with Abcam ab64186 pAb revealed that the intensity of the ∼34 kDa band was reduced from cells transfected with NRN1‐targeted siRNA, compared to scramble controls (Figure 4B). However, the ∼150‐kDa band was not observed in these experiments. Collectively, these results suggest that Abcam ab64816 pAb does not yield spurious bands from HEK293T or SH‐SY5Y cells, which lack endogenous NRN1, but can potentially detect endogenous NRN1 protein from N2a cells with a degree of specificity.

### 3.5 NRN1 protein levels in PSP and CBD brains

**FIGURE 5:**
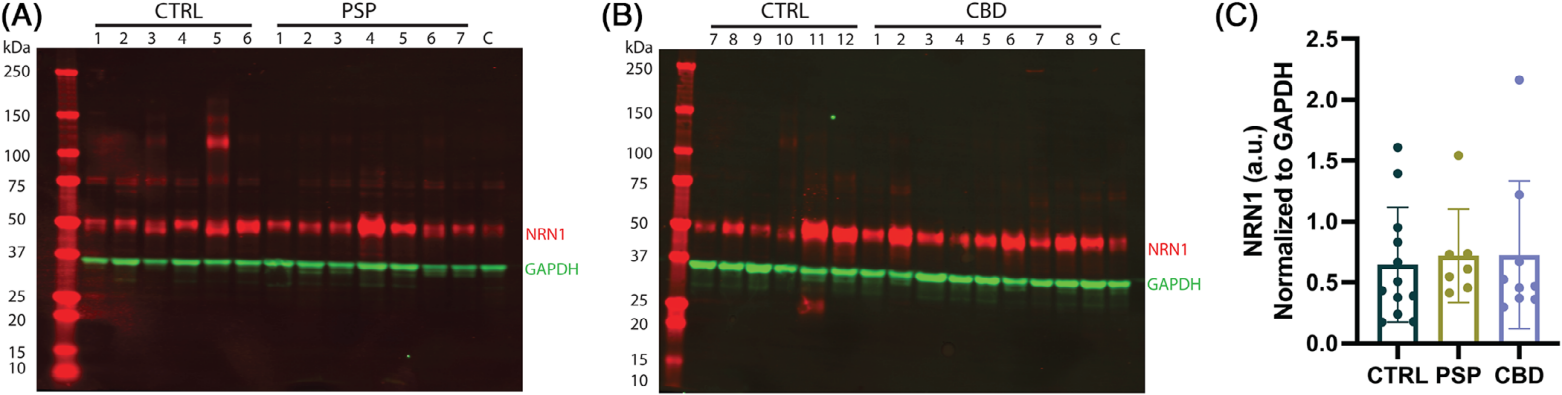
NRN1 protein levels in prefrontal cortex of human tauopathy cases. (A and B) Representative Western blots of control (CTRL), progressive supranuclear palsy (PSP), and corticobasal degeneration (CBD) cases (50 µg protein), probed with NRN1 polyclonal antibody Abcam ab64186. “C” indicates a control sample loaded on each Western blot to compare densitometry across blots. Densitometry analysis of (A) and (B) reveals that NRN1 levels are comparable across cases when normalized to GAPDH. One‐way ANOVA, *F*(2,25) = 0.08148, *p* = 0.9220, with Tukey's multiple comparisons test. Error bars represent standard deviation of the mean. a.u. = arbitrary units; GAPDH, glyceraldehyde‐3‐phosphate dehydrogenase; NRN1, Neuritin‐1;.

Past studies provided evidence that NRN1 mRNA levels were reduced in the hippocampus of 6‐month‐old Tg2576 APP transgenic mice when compared to WT NTG mice.^21^ These studies support the hypothesis that Aβ contributes to the reduction of NRN1 protein abundance in AD.^9^, ^18^ However, determining whether tau plays a role in the reduction of NRN1 protein abundance is an important question. Therefore, we sought to test whether NRN1 protein levels were altered in primary tauopathy cases, where tau was the major contributing factor to the neurodegenerative process.^49^ We evaluated PSP and CBD cases (Table S1). Age‐ and sex‐matched *post mortem* dorsolateral prefrontal cortex (DLPFC) tissues from control (*n* = 12), PSP (*n* = 7), and CBD (*n* = 9) cases were selected for comparison from the Emory University Alzheimer's Disease Research Center brain bank. PSP and CBD cases underwent extensive neuropathological characterization required for diagnosis based on established criteria.^29^, ^30^ DLPFC whole homogenate tissue from all cases was used for Western blotting and densitometry analysis, with a control sample (C) loaded on each Western blot to compare densitometry across blots. Notably, Abcam ab64186 pAb detected NRN1 protein levels at ∼50 kDa in all human cases (Figure 5A,B). Densitometry analysis revealed that NRN1 protein levels were comparable across control, PSP, and CBD cases (Figure 5C). However, a limitation of this analysis is the relatively small sample size of the PSP cases, which reduces the statistical power and generalizability of the findings. Based on this, we posit that tau pathology does not affect NRN1 protein abundance in the DLPFC of PSP and CBD cases. However, these findings do not exclude the possibility that tau influences NRN1 protein levels in other tauopathies, including AD.

### 3.6 NRN1 levels in PS19 tauopathy mice

**FIGURE 6:**
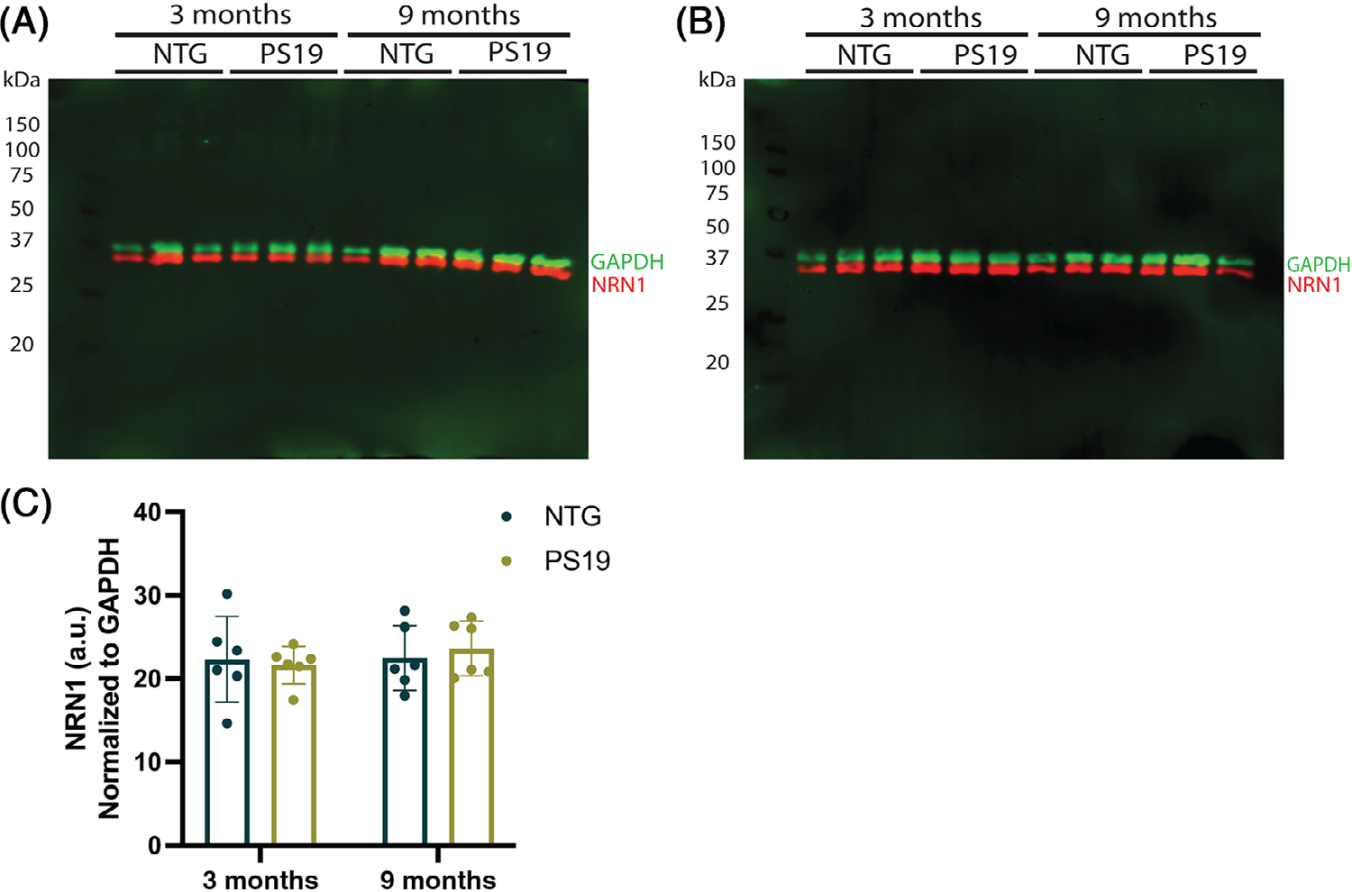
NRN1 protein levels in prelimbic medial prefrontal cortex of PS19 mice. (A and B) Representative Western blots of 3‐ and 9‐month NTG and PS19 mice (50 µg protein), probed with NRN1 polyclonal antibody Abcam ab64186 and GAPDH monoclonal antibody. Densitometry analysis of panels (A) and (B) reveals that NRN1 protein levels in NTG and PS19 mice are comparable at 3 and 9 months. Two‐way ANOVA (*F*[1, 20] = 0.3, *p *= 0.5633) with Tukey's multiple comparisons test. Each point represents one mouse. *N* = 6 NTG mice at 3 months (six male [M], six female [F]), six PS19 mice at 3 months (six M, six F), six NTG mice at 9 months (six M, six F), six PS19 mice at 9 months (6 M, 6 F). Error bars: standard deviation of the mean. a.u. = arbitrary units; GAPDH, glyceraldehyde‐3‐phosphate dehydrogenase; NRN1, Neuritin‐1; NTG, non‐transgenic.

As a secondary assessment of tauopathy influence on endogenous NRN1 protein abundance, we evaluated the Tau P301S mouse line (PS19), a commonly used tauopathy model.^31^, ^50^, ^51^, ^52^, ^53^ NFT‐like tau inclusions have been reported in the prelimbic mPFC of PS19 by 3 months of age, with robust hyperphosphorylated AT8^+^ tau in the mPFC at 9 months of age.^31^, ^54^ Based on this, age‐ and sex‐matched PS19 and NTG mice were used to evaluate whether accumulation of tau pathology influenced NRN1 protein levels. Whole homogenate tissue from the mPFC of 3‐ or 9‐month‐old PS19 and NTG mice was used for Western blot analysis. Abcam ab64186 pAb detected NRN1 protein levels at ∼34 kDa, in both PS19 and NTG mice, consistent with our previous analysis of WT C57BL/6N mice (Figure 6A,B). Densitometry analysis indicated that endogenous NRN1 protein levels were comparable in PS19 and NTG littermates at 3 and 9 months of age (Figure 6C). These results further support the hypothesis that tauopathy in the absence of Aβ is not sufficient to induce a reduction of NRN1 protein level.

### 3.7 NRN1 in cultured human ioGlutamatergic neurons

**FIGURE 7:**
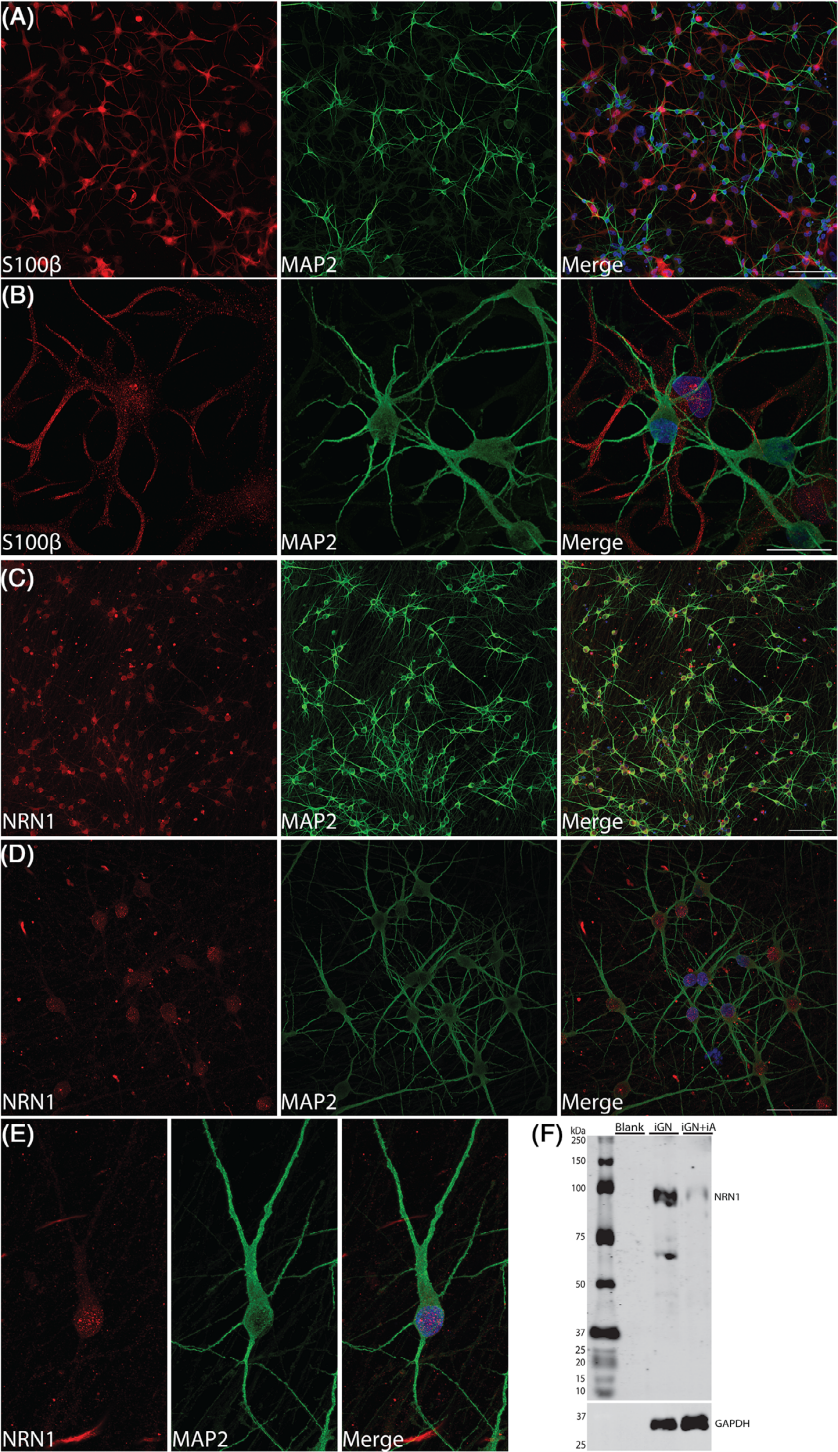
NRN1 protein in human ioGlutamatergic neurons. (A–E) Representative confocal microscopy images of cultured ioGlutamatergic neurons and ioAstrocytes. DAPI in blue. (A and B) S100β (red) identifies ioAstrocytes and MAP2 (green) identifies ioGlutamatergic neurons at 20× (scale bar = 100 µm) (A) and 60× zoom = 2 (1024 × 1024) (scale bar = 25 µm) (B). (C–E) NRN1 (red) and MAP2 (green) at 20× (scale bar = 100 µm) (C), 60× (1024 × 1024) (scale bar = 50 µm) (D), and 60× zoom = 2 (1024 × 512) (E). (F) Representative Western blot of ioGlutamatergic neurons cultured in isolation (iGN) and co‐culture of ioGlutamatergic neurons and ioAstrocytes (iGN+iA), probed with NRN1 polyclonal antibody Abcam ab64186 and GAPDH monoclonal antibody. First lane of the representative blot was left blank, and 25 µg protein was loaded per lane. GAPDH was probed as a loading control. GAPDH, glyceraldehyde‐3‐phosphate dehydrogenase; NRN1, Neuritin‐1.

To test for the presence of NRN1 in human neurons that were programmed from human induced pluripotent stem cells (iPSCs), ioGlutamatergic neurons and ioAstrocytes were purchased from bit.bio and cultured following bit.bio protocols. ioGlutamatergic neurons and ioAstrocytes were co‐cultured on glass coverslips for 23 days, then fixed and imaged by confocal microscopy. Immunoreactivity to S100β or MAP2, identified ioAstrocytes or ioGlutamatergic neurons, respectively (Figure 7A,B). NRN1 immunoreactivity displayed a speckled pattern that was localized to somas of MAP2‐positive ioGlutamatergic neurons (Figure 7C), as well as surrounding nuclei of MAP2‐positive ioGlutamatergic neurons (Figure 7D,E). Notably, NRN1 immunoreactivity from Abcam ab64186 pAb also displayed an aberrant, potentially non‐specific signal that was localized outside of cells (Figure 7C–E).

NRN1 protein was observed consistently at ∼34 kDa in primary rodent cortical neurons and mouse brain, and in Figure 5, NRN1 protein was detected predominantly at ∼50 kDa in DLPFC samples of human cases. To test whether the NRN1 protein size from human brain was consistent with human neurons in culture, ioGlutamatergic neurons were grown in isolation or in co‐culture with ioAstrocytes. At DIV 23, cells were harvested for Western blot analysis. Abcam ab64186 pAb detected a band at ∼95 kDa from ioGlutamatergic neurons and the same band from co‐cultured lysates, albeit at reduced intensity (Figure 7F). Likely, the reduction in NRN1 signal from co‐cultured lysates is that NRN1 is expressed at an extremely low level or not at all in ioAstrocytes. The presence of faint bands at ∼70 and 45 kDa from ioGlutamatergic neurons may reflect other NRN1 isoforms with post‐translational modifications, macromolecular protein combinations of NRN1 monomers, or non‐specific bands.

## 4 DISCUSSION

Individuals who maintain cognitive function despite high levels of AD‐associated pathology appear resilient in life to clinical manifestations of dementia.^55^ These individuals represent a clinically interesting subset of the population, and we hypothesized that such individuals would exhibit cognitive resilience that conferred the ability to maintain cognitive function despite the accumulation of AD‐related pathologies. Unbiased proteomics have identified multiple cortical proteins strongly associated with levels of resilience, including NRN1.^18^ We previously identified NRN1 as a hub protein that co‐expressed with other synaptic proteins that remained increased in resilient individuals compared to symptomatic AD cases.^9^ We expanded this analysis herein by evaluating NRN1 using the TREAT‐AD risk score assessment. Through this mechanism, we determined that the decreased expression level of NRN1 mRNA and protein abundance in AD brains relative to controls was likely due to the reduction of NRN1 expression in excitatory neurons over the course of AD progression. Moreover, NRN1's candidacy as a therapeutic target was further supported by our re‐evaluation of how NRN1 affects the proteome of cultured neurons. This showed that NRN1 could reverse the Synapse domain proteomic signatures that are present in AD brains. However, the potential for differential response between rat primary neurons and the human brain is an important limitation to this comparative analysis. While proteomics is a powerful tool for identifying relatively broad biological pathways for therapeutic intervention, few studies have named individual molecules as potential therapeutic targets and demonstrate functional validation of target proteins. This is, in part, due to the lack of reliable tools to probe, measure, and perturb target protein structures.

Here, we reported the characterization of a commercially available polyclonal (pAb) NRN1 antibody in in vitro and in vivo model systems as well as *post mortem* human brain samples. We compared three commercially available pAb NRN1 antibodies, Invitrogen PA5‐47406, Bioss bs‐2464R, and Abcam ab64186, for Western blotting analyses. Invitrogen PA5‐47406 pAb was not reported previously and did not detect endogenous NRN1 protein in our samples. Wang et al. demonstrated that Bioss bs‐2464R detected endogenous NRN1 protein expression in primary rat hippocampal neurons; however, Bioss bs‐2464R did not detect endogenous NRN1 protein in our cortical or hippocampal cultures.^56^ Moreover, Wang et al. did not report an observed molecular weight for NRN1. Abcam ab64186 pAb was reported previously to detect NRN1 protein in U251 human astrocytoma cell lines and in endometriotic tissue collected from patients with endometriosis.^43^, ^44^ Feng et al. did not report an observed molecular weight of the NRN1 protein; however, Rahmawati et al. reported that the NRN1 protein in endometriotic tissue was approximately 15 kDa, consistent with the predicted size of the full‐length protein.^20^ A previous study reported that Abcam ab64186 pAb could detect NRN1 protein expression in the hippocampus of WT mice at 1, 4, and 7 months, but the observed molecular weight was not reported.^57^ In the present study, Abcam ab64186 pAb detected endogenous NRN1 protein at ∼34 kDa in primary cultured neuron lysates, mouse brains, and N2a murine cells. Endogenous NRN1 protein was detected in murine N2a cells but not in human SH‐SY5Y cells. This may be attributed to species‐specific transcriptional regulation and/or differences in cell blastoma subtype and origin. N2a cells were derived from mouse neuroblastoma,^58^ whereas SH‐SY5Y cells originated from a metastatic bone tumor biopsy.^59^, ^60^ SH‐SY5Y cells harbor the potential for additional experimental differentiation to a more neuron‐like fate, and under those conditions, it could lead to endogenous expression of NRN1.^60^

Previous reports suggested NRN1 might exist in two forms, membrane‐bound and soluble; however, the physiological functions of each form remain unknown. The NRN1 gene encodes a small, highly conserved protein containing a secretory signal sequence at the N‐terminus and a consensus sequence for glycosylphosphatidylinositol (GPI) at the C‐terminus.^20^ This GPI linkage enables NRN1 to anchor at cell surfaces, and upon cleavage of GPI by phospholipase, the resultant soluble NRN1 is released into the extracellular space.^20^, ^61^ Past studies provided evidence that NRN1 existed predominantly as the soluble form in vivo and exerts neurotrophic effects on synaptic maintenance and neuronal survival.^20^, ^62^, ^63^ We hypothesized that the mature, secreted form of NRN1 would have a molecular weight of ∼15 kDa, and the ∼34 kDa band observed in primary cultured neuron lysates, mouse brains, and N2a cells would reflect the dimerization of NRN1 into its mature, biologically active form. Members of the neurotrophic family, including BDNF and neurotrophin‐3 (NT‐3), are known to function as non‐covalent homodimers in their mature, biologically active forms.^46^ Dimerization is essential for receptor binding, activation, and downstream signaling pathways that impact neuronal growth, survival, and plasticity. Moreover, identification of NRN1 at ∼34 kDa corroborated previous results that NRN1 could form homodimers in vitro.^64^ In N2a cells, NRN1 was observed at molecular weights of ∼34 and ∼150 kDa. The ∼150‐kDa bands may reflect macromolecular NRN1 protein complexes but could also be interpreted as NRN1 aggregation and/or highly abundant post‐translational modifications. It is also possible that the ∼150‐kDa bands are non‐specific bands from the Abcam ab64186 pAb and exclusive to N2a cells. Further studies are needed to elucidate the distinctions between GPI‐anchored, secreted, and dimeric NRN1. In immunocytochemistry experiments involving human glutamatergic neurons, the Abcam ab64186 pAb displayed noticeable immunoreactivity that was not associated with cells and likely not representative of NRN1 (Figure 7C–E). These potentially non‐specific signals could yield false positives if Abcam ab64186 pAb were used to explore NRN1 in human or mouse brain sections by immunohistochemistry (IHC). However, if a more suitable NRN1 antibody was identified or generated for IHC studies, questions regarding the presence of extracellular versus intracellular NRN1 could be addressed in brain tissue as well as surveys of NRN1 protein abundance across regions.

Notably, we observed NRN1 at ∼50 kDa in *post mortem* human brain samples and at ∼95 kDa in human glutamatergic neurons. The disconnect in observed molecular weight of NRN1 in human brain and cultured human neurons may reflect the differentiation state of neurons that are expressing NRN1. For instance, all *post mortem* human brain samples were from aged individuals, whereas the cultured neurons were in a highly immature state. Moreover, it is possible that NRN1 has different functional roles at various times in the human lifespan that require particular formations of NRN1 protein complexes. This hypothesis assumes that the ∼95‐kDa band from cultured glutamatergic neurons represents multiplex NRN1 monomers, whereas it is possible the ∼95‐kDa band indicates NRN1 protein aggregation or additional post‐translational modifications. The human NRN1 gene (candidate plasticity gene 15, *CPG15*) is highly conserved across vertebrate species, with a homology of approximately 97% between human and mouse/rat.^20^, ^45^ Fujino et al. identified the only paralogue of *CPG15* in the mouse and human genome, *CPG15‐2* (i.e., *NRN1L*). Within the central nervous system, *CPG15* mRNA is most abundant in the cerebral cortex, followed by the hippocampus, whereas *CPG15‐2* mRNA is most abundant in the retina, followed by the olfactory bulb.^64^ However, to date, additional studies regarding the role of *CPG15‐2* are lacking. Currently, there are no studies that have reported the potential post‐translational modifications that may lead to an increase in the molecular weight of NRN1 in humans^65^ provided evidence of potential ubiquitin‐mediated regulation of NRN1, although this study does not directly characterize post‐translational modifications on NRN1 itself. Further studies are needed to interrogate putative post‐translational modifications on NRN1.

Previous studies investigated the potential therapeutic use of NRN1 against Aβ‐induced synaptic deficits in cultured neurons and Tg2576 APP transgenic mice.^9^, ^21^, ^66^, ^67^ revealed that mRNA levels of NRN1 decreased significantly in the hippocampus of 6‐month‐old Tg2576 transgenic mice when compared to WT mice. Furthermore, infusion of recombinant NRN1 into Tg2576 transgenic mice rescues deficits in hippocampal long‐term potentiation in the Schaffer collateral pathway.^66^ Consistent with this study, Choi et al.^66^ observed that viral‐mediated overexpression of NRN1 in the dentate gyrus of 13‐month‐old Tg2576 mice attenuated deficits in learning and memory. Our past studies revealed that NRN1 could facilitate dendritic spine resilience against Aβ and block Aβ‐induced neuronal hyperexcitability.^9^ However, in order to support NRN1 as a rational therapeutic target to promote resilience and delay dementia onset, it is imperative to assess whether NRN1 can provide neuroprotection against tau. A recent omics study identified NRN1 as a commonly differentially expressed gene in NFT‐bearing neurons within chemical synaptic transmission pathways.^68^ Our results herein suggest that tau pathology alone may not alter NRN1 protein abundance in the dorsolateral prefrontal cortex. However, since we only measured protein abundance, the data do not assess potential changes to NRN1 function, localization, or post‐translational modifications. It is important to note that the exact brain region(s) responsible for conferring enhanced resilience remain unclear. We selected the prefrontal cortex based on previous proteomic findings.^18^ Our results do not exclude the possibility that NRN1 protein levels may be altered in brain regions more susceptible to tau‐related pathology in AD (i.e., entorhinal cortex).^69^

One general hypothesis in the field is that pathological tau induces synapse silencing without causing overt destruction of dendritic structure. Silent synapses lack functional α‐amino‐3‐hydroxy‐5‐methyl‐4‐isoxazolepropionic acid receptors (AMPARs), rendering the synapse inactive.^70^, ^71^ The vulnerability of AMPARs to tau pathology can drive synaptic dysfunction in age‐related tauopathies.^72^, ^73^, ^74^ Previous studies showed that NRN1 promoted synapse maturation through the recruitment of AMPARs to the postsynapse.^23^ Moreover, a recent quantitative proteomic study identified NRN1 as a novel protein constituent of AMPAR complexes.^75^ Importantly, it has been revealed that NRN1 interacts directly with the GluA1 AMPAR subunit and is critical for recruitment of PSD95 to AMPAR‐containing spines.^27^ Thus, further mechanistic studies are needed to determine whether NRN1 can mitigate pathogenic tau levels and synaptic deficits.

## CONFLICT OF INTEREST STATEMENT

The authors declare no conflicts of interest. Author disclosures are available in the Supporting Information.

## CONSENT STATEMENT

For human subject research, de‐identified human samples and data were used in this study. Consent was not necessary.

## Supporting information



Supporting Information

Supporting Information
