# Supplementary material for: NRN1 as a therapeutic target for Alzheimer's disease
Source: Alzheimers Dement. 2026 Feb 6;22(2):e71149. doi: 10.1002/alz.71149 (PMC12877949; doi:10.1002/alz.71149)
Supplement: Supplementary file 1 — Supporting Information [file ALZ-22-e71149-s002.docx]

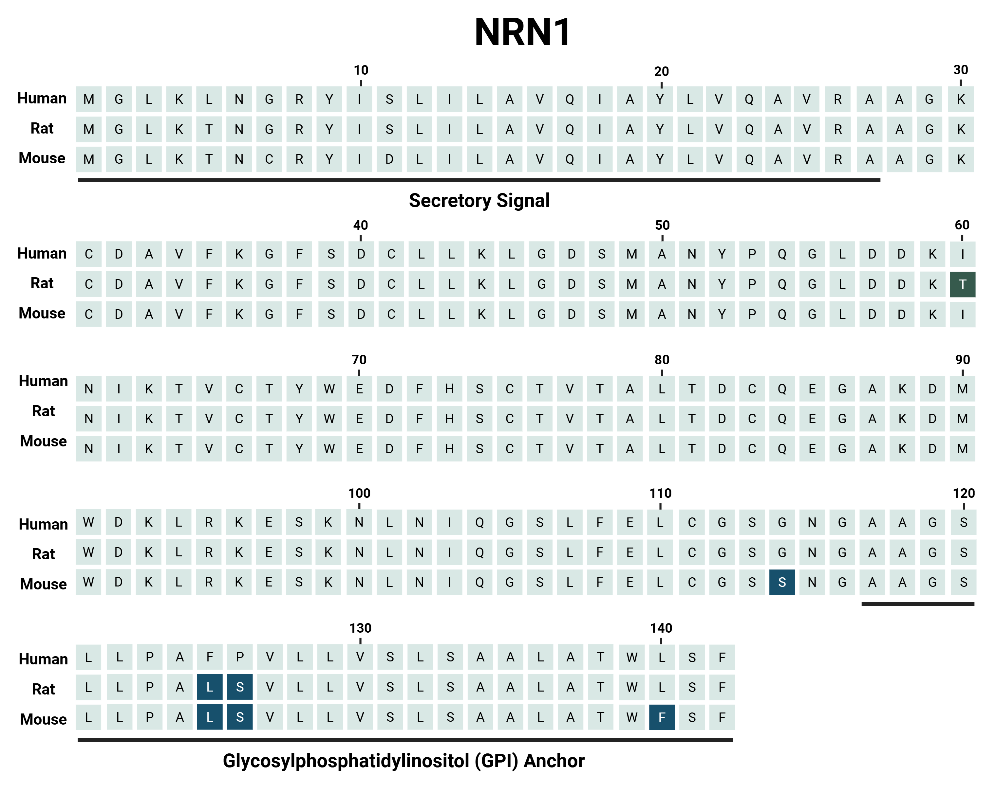


**Supporting Information 1. Multiple sequence alignment of human, rat, and mouse NRN1 protein.** The full amino acid sequences of human, rat, and mouse NRN1 are shown. Only the amino acids that differ from human are highlighted in dark green. The predicted signal peptide for secretion and GPI anchor are underlined.

**
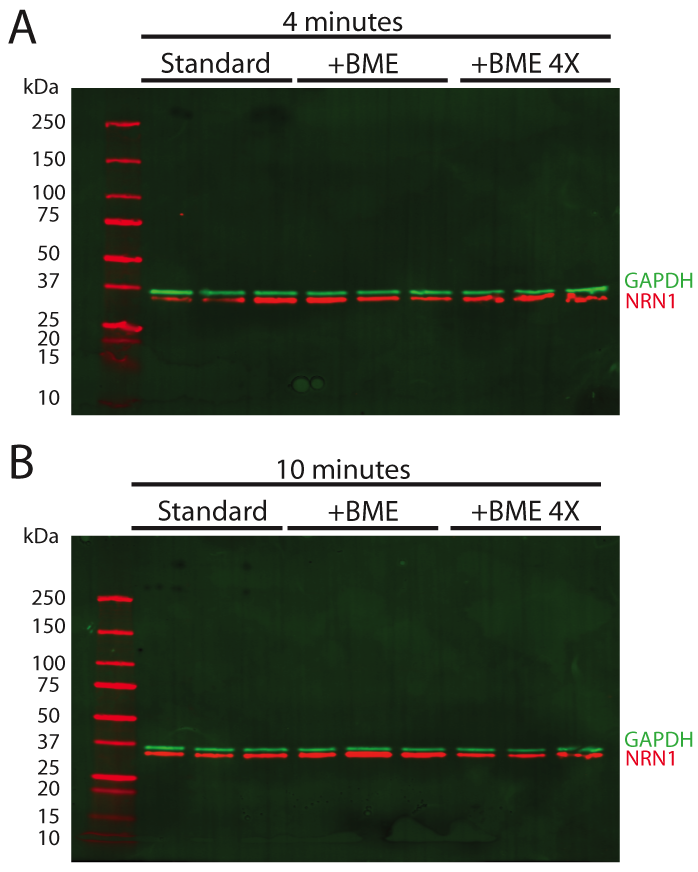
**

**Supporting Information 2. NRN1 protein under denaturing conditions with BME. (A-B)** Representative Western blot of rat primary cortical neuron lysates, probed with NRN1 polyclonal antibody Abcam ab64186. Lysates were treated with fresh preparations of 2-Mercaptoethanol (BME), then boiled at 95ºC for 4 (A) or 10 min (B), respectively. Standard indicates loading dye used to load samples into each well. BME indicates the addition of freshly prepped BME added to the loading dye used to load samples into each well. BME 4X indicates four times the concentration of freshly prepped BME used to load samples into each well. N=3.


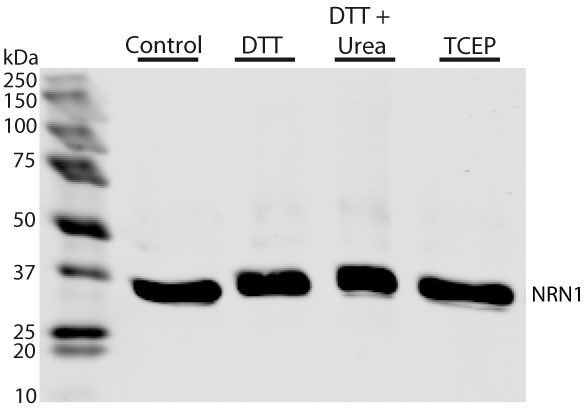


**Supporting Information 3. NRN1 protein under denaturing conditions with DTT, Urea, or TCEP.** Representative Western blot of rat primary cortical neuron lysates, probed with NRN1 polyclonal antibody Abcam ab64186. Lysates were treated with fresh preparations of dithiothreitol (DTT), DTT and Urea, or Tris (2-carboxyethyl) phosphine (TCEP), then boiled at 95ºC for 4 min. Control indicates loading dye used to load samples into each well. DTT indicates the addition of freshly prepped DTT 50 mM added to the loading dye used to load samples into each well. DTT + Urea indicates the addition of freshly prepped DTT 50 mM and 8M Urea used to load samples into each well. TCEP indicates the addition of freshly prepped TCEP 50 mM added to the loading dye used to load samples into each well. 10 µg of protein lysate were loaded per lane. N=3.

| **Table S1. Human Cases** |  |  |  |  |
| --- | --- | --- | --- | --- |
| Case | Race/Sex | Age at onset | Age at death | PMI (h) |
| Control (*n* = 12) |  |  |  |  |
| 1 | W/F |  | 46 | 6.5 |
| 2 | B/M |  | 53 | 6.5 |
| 3 | B/F |  | 57 | 17 |
| 4 | W/M |  | 57 | 10 |
| 5 | W/M |  | 58 | 6 |
| 6 | B/F |  | 60 | 8 |
| 7 | W/M |  | 65 |  |
| 8 | W/M |  | 66 | 10 |
| 9 | H/M |  | 70 | 4.5 |
| 10 | W/F |  | 74 | 3 |
| 11 | B/F |  | 68 | 11 |
| 12 | W/F |  | 92 | 15.5 |
| PSP (*n* = 7) |  |  |  |  |
| 1 | W/M | 51 | 61 | 3 |
| 2 | W/F | 61 | 70 | 15 |
| 3 | W/M | 65 | 75 | 10 |
| 4 | W/F | 74 | 82 | 11.5 |
| 5 | W/M | 70 | 83 | 10 |
| 6 | W/M | 77 | 83 | 23 |
| 7 | W/F | 89 | 94 | 14 |
| CBD (*n* = 9) |  |  |  |  |
| 1 | W/M | 33 | 44 | 24 |
| 2 | H/M | 56 | 61 | 2.75 |
| 3 | W/M | 57 | 62 | 5 |
| 4 | W/M | 53 | 65 | <8 |
| 5 | W/F | 63 | 68 | 3 |
| 6 | W/F | 72 | 78 | 8.5 |
| 7 | W/F | 72 | 82 | 3 |
| 8 | W/F | 78 | 86 | 3 |
| 9 | M |  | 91 | 24 |
| Case numbers correspond to immunoblot samples in Figure 6. If values are blank, information was unavailable. PSP = progressive supranuclear palsy, CBD = corticobasal degeneration. PMI = Postmortem interval. Race: W = White, A = African American, H= Hispanic. Sex: F= Female; M = Male. | | | | |
